# Neuroinflammation and Natural Antidepressants: Balancing Fire with Flora

**DOI:** 10.3390/biomedicines13051129

**Published:** 2025-05-07

**Authors:** Ana Clara Figueiredo Godoy, Fernanda Fortes Frota, Larissa Parreira Araújo, Vitor E. Valenti, Eliana de Souza Bastos Mazuqueli Pereira, Claudia Rucco P. Detregiachi, Cristiano M. Galhardi, Flávia Cristina Caracio, Rafael S. A. Haber, Lucas Fornari Laurindo, Masaru Tanaka, Sandra M. Barbalho

**Affiliations:** 1 Department of Biochemistry and Pharmacology, School of Medicine, Universidade de Marília (UNIMAR), Marília 17525-902, SP, Brazillarissaparreiraaraujo@gmail.com (L.P.A.);; 2 Autonomic Nervous System Center, School of Philosophy and Sciences, São Paulo State University, Marília 17525-900, SP, Brazil; 3 Postgraduate Program in Structural and Functional Interactions in Rehabilitation, School of Medicine, Universidade de Marília (UNIMAR), Marília 17525-902, SP, Brazillucaslaurindo@unimar.br (L.F.L.); 4 School of Medicine, Faculdade de Medicina de Marília (FAMEMA), Marília 17519-030, SP, Brazil; 5 Danube Neuroscience Research Laboratory, HUN-REN-SZTE Neuroscience Research Group, Hungarian Research Network, University of Szeged (HUN-REN-SZTE), Tisza Lajos krt. 113, H-6725 Szeged, Hungary; 6 Research Coordinator at UNIMAR Charity Hospital, Marília 17525-902, SP, Brazil

**Keywords:** major depressive disorder (MDD), plant-based therapeutics, neuroinflammation, oxidative stress, natural antidepressants, tryptophan, kynurenine, brain-derived neurotrophic factor (BDNF), mitochondrial dysfunction, curcumin

## Abstract

**Background/Objectives**: Major depressive disorder (MDD) is a major global health concern that is intimately linked to neuroinflammation, oxidative stress, mitochondrial dysfunction, and complicated metabolic abnormalities. Traditional antidepressants frequently fall short, highlighting the urgent need for new, safer, and more acceptable therapeutic techniques. Phytochemicals, i.e., natural antidepressants derived from plants, are emerging as powerful plant-based therapies capable of targeting many pathogenic pathways at the same time. **Summary**: This narrative review synthesizes evidence from preclinical and clinical studies on the efficacy of phytochemicals such as curcumin, polyphenols, flavonoids, and alkaloids in lowering depressed symptoms. Consistent data show that these substances have neuroprotective, anti-inflammatory, and antioxidant properties, altering neuroimmune interactions, reducing oxidative damage, and improving mitochondrial resilience. Particularly, polyphenols and flavonoids have great therapeutic potential because of their capacity to penetrate the blood–brain barrier, inhibit cytokine activity, and encourage neuroplasticity mediated by brain-derived neurotrophic factor (BDNF). Despite promising results, the heterogeneity in study designs, phytochemical formulations, and patient demographics highlights the importance of thorough, standardized clinical studies. **Conclusions**: This review identifies phytochemicals as compelling adjuvant or independent therapies in depression treatment, providing multimodal mechanisms and enhanced tolerability. Additional research into improved dosage, pharmacokinetics, long-term safety, and integrative therapy approaches is essential. Using phytotherapeutics could considerably improve holistic and customized depression care, encouraging new research routes in integrative neuroscience and clinical psychiatry.

## 1. Introduction

**Figure 1:**
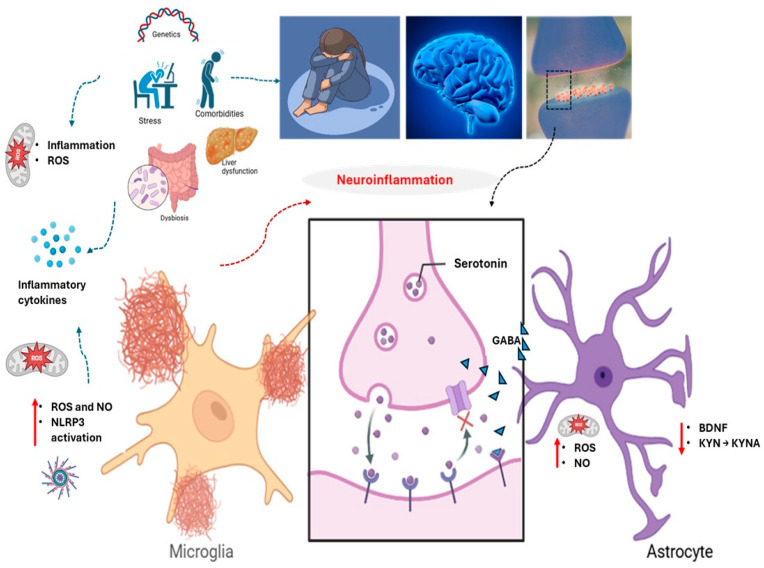
Neurobiological contributors to the onset and progression of major depressive disorder (MDD). Multiple interacting systems—including genetic predisposition, chronic psychosocial stress, systemic comorbidities, and immune dysregulation—converge to disrupt neuroimmune homeostasis. Central to this pathophysiology are oxidative stress, liver dysfunction, intestinal dysbiosis, and neuroinflammation, driven by activated microglia and astrocytes. These cells increase reactive nitrogen and oxygen species (NO and ROS), impair mitochondrial function, and alter kynurenine (KYN) pathway dynamics, reducing kynurenic acid (KYNA) and promoting neurotoxicity. Dysregulated serotonin and gamma-aminobutyric acid (GABA) signaling, alongside diminished brain-derived neurotrophic factor (BDNF), further impair synaptic plasticity and emotional regulation. ↑: increase; ↓: decrease.

Major depressive disorder (MDD) is a common and debilitating mental condition characterized by persistent low mood, anhedonia, cognitive dysfunction, and severe impairments in occupational, social, and interpersonal functioning [1]. Aside from its impact on quality of life, MDD remains the leading psychiatric contributor to global suicide mortality, with core symptoms including diminished self-worth, excessive guilt, psychomotor changes, and sleep disturbances—factors that collectively contribute to increased all-cause mortality rates [2]. Patients with MDD often show persistent negative cognitive biases, social withdrawal, and impaired emotional regulation [3]. Epidemiological data reveal an increasing global burden, presently impacting over 350 million individuals, with World Health Organization forecasts indicating that MDD will emerge as the foremost cause of disability worldwide by 2030 [4,5]. Vulnerable populations, such as pregnant women, the elderly, and children, have disproportionately high incidence rates, emphasizing the role of psychological stressors, genetic predisposition, and environmental adversity in its etiology [6,7,8,9]. Chronic stress, in particular, has been linked to the pathophysiology of MDD because of its ability to disrupt neuroplastic processes, resulting in neuronal atrophy, synaptic loss, and volumetric changes in key brain regions such as the hippocampus and prefrontal cortex, all of which contribute to the disorder’s affective, cognitive, and behavioral symptoms [10,11,12]. Figure 1 summarizes the primary neurobiological mechanisms involved in MDD pathogenesis. The purpose of this state-of-the-art critical narrative review is to integrate disparate mechanistic, pre-clinical, and clinical data into a coherent conceptual model of phytochemical action in depression rather than to catalogue every published study.

Emerging evidence underscores the critical role of neuroinflammatory processes in the pathogenesis of MDD, positioning central nervous system (CNS) inflammation as a key mechanistic driver in a subset of patients [13]. A growing body of research implicates dysregulation within the kynurenine pathway of tryptophan metabolism—a principal biosynthetic route for serotonin—in contributing to both decreased serotonergic tone and increased production of neurotoxic metabolites [14]. These metabolic changes not only disrupt neurotransmitter homeostasis, but they also increase susceptibility to mood dysregulation and cognitive impairment, which are hallmarks of depressive pathology [15,16,17,18]. Furthermore, metabolomic profiling investigations in MDD patients regularly reveal abnormalities in a variety of circulating metabolites, including tryptophan, tyrosine, methionine, valine, phenylalanine, pyruvate, kynurenic acid, and deoxycholic acid [19]. These metabolic disruptions are closely associated with impaired neuroprotective mechanisms, increased oxidative burden, dysregulated apoptotic signaling, and chronic low-grade inflammation [20,21,22]. Collectively, these pathophysiological alterations foster a neurobiological environment conducive to the initiation and persistence of depressive symptoms, particularly through the destabilization of neuronal resilience and synaptic integrity [23].

While traditional pharmacological treatments, particularly tricyclic antidepressants (TCAs) such as imipramine, amitriptyline, clomipramine, desipramine, and doxepin, remain essential in the treatment of MDD, their therapeutic efficacy is primarily mediated by modulation of monoaminergic neurotransmission, specifically by increasing synaptic concentrations of serotonin and norepinephrine [4]. Despite their clinical value, emerging evidence suggests that while antidepressants are considered first-line therapies, they are not the only effective modality for managing MDD [2]. Notably, in pediatric and adolescent populations, non-pharmacological strategies such as psychological support, scheduled physical activity, and dietary optimization have shown clinically relevant preventative and therapeutic outcomes [24]. Nonetheless, a large proportion of people show partial or complete resistance to these therapies, emphasizing the importance of complementary or alternative therapy approaches [25]. Phytotherapeutics have recently rekindled scientific attention due to their historical use, biological plausibility, cost effectiveness, and acceptable side-effect profiles. Medicinal plants have historically provided bioactive chemicals with neuroprotective, anti-inflammatory, and neuromodulatory effects [26]. These natural compounds persist in guiding the discovery and development of innovative pharmacological medicines, presenting a potentially beneficial adjunctive strategy for persons with treatment-resistant or recurrent depression [27,28].

Phytochemicals are naturally occurring bioactive molecules present in a variety of plants and food sources that influence their pigmentation, fragrance, and flavor profiles [29]. These chemicals, which include alkaloids, flavonoids, steroids, coumarins, and terpenoids like eucalyptol, are increasingly recognized for their therapeutic effects, particularly in situations characterized by oxidative stress and chronic inflammation [30]. Aside from their well-established antioxidant and anti-inflammatory properties, phytochemicals also have vasoprotective and cardiometabolic effects, making them prospective agents in the prevention and treatment of complex, multifactorial illnesses [31]. Phytocompounds have received a lot of attention in neuropsychiatry because of their possible role in altering important neurobiological processes involved in neurodegeneration and mood disorders. Compounds derived from plants such as *Aizoaceae*, *Acorus*, *Korthalsella*, *Astragalus membranaceus*, *Sophora flavescens*, and *Ononis spinosa* have long been employed in traditional medicine systems for their psychotropic and neuromodulatory effects [32,33,34]. Preclinical research and ongoing clinical trials indicate that several of these botanicals have antidepressant-like properties via processes involving monoaminergic regulation, neurotrophic signaling, and neuroinflammatory cascade reduction [35]. As a result, the incorporation of phytocompounds into therapeutic frameworks for MDD is increasingly being investigated both as standalone treatments and as adjuncts to traditional pharmacotherapy [36]. Their ability to address many pathogenic domains at once makes them especially useful in the context of MDD, which has complex and heterogeneous neuronal bases [37].

It is well established that neurodevelopment intricately shapes emotional regulation and cognitive function, both of which are highly susceptible to disruption by psychiatric and neurological disorders [38]. In recent decades, the global burden of neuropsychiatric conditions—particularly those with complex, multifactorial etiologies such as MDD—has escalated significantly, placing substantial pressure on healthcare systems and highlighting the urgent need for innovative, accessible, and well-tolerated therapeutic strategies [39,40,41]. Although conventional antidepressants remain central to current treatment paradigms, their limitations—namely delayed onset of action, treatment resistance, and undesirable side-effect profiles—have driven the search for alternative and adjunctive interventions that can engage broader neurobiological targets with improved safety margins [42,43].

In this regard, phytocompounds have emerged as intriguing candidates due to their pleiotropic modes of action, which include anti-inflammatory, antioxidant, and neuromodulatory capabilities [44]. Recent research has highlighted the intricate connection between phytochemicals, neuroinflammation, and mitochondrial health [22,45]. Yet, no previous analysis has definitively identified the clinical trial landscape assessing the effectiveness of general phytocompounds in MDD despite growing preclinical evidence and sporadic clinical research suggesting their potential usefulness. Notably, recent reviews contend that not all trials have yielded favorable outcomes, reinforcing the need for further research to clarify underlying mechanisms, optimize dosage regimens, assess long-term safety, and determine the comparative effectiveness of medicinal plants in the treatment of depression [46]. Addressing this gap, this comprehensive narrative review seeks to illuminate the diverse roles that phytotherapeutics may play in depression management. We hope these insights will not only guide future investigations on optimal usage and mechanisms of action but also encourage the meaningful integration of plant-based interventions into established, evidence-based psychiatric care.

We compiled an **expert-guided evidence map**: senior authors snowballed references from seminal phytochemical-depression papers and maintained weekly PubMed, Scopus, and Web of Science keyword alerts (“phytochemical* AND depression OR MDD”) through 28 February 2025. Records were double-screened and retained only when they advanced mechanistic understanding or held translational relevance, deliberately privileging depth over exhaustive coverage. This theory-driven selection underpins the narrative synthesis while avoiding the rigidities of systematic-review protocols. Our theory-driven, expert-guided evidence map inevitably introduces selection bias; some pertinent studies may therefore be absent. This trade-off was accepted to prioritize analytic depth over exhaustive coverage. To improve clarity and avoid redundancy, overlapping descriptions of key mechanisms such as oxidative stress, mitochondrial dysfunction, and neuroinflammation were streamlined. These interconnected processes are herein discussed in focused sections with minimal repetition, ensuring conceptual continuity without diluting analytical depth.

## 2. Neuroinflammation: A Core Driver of Severe Depression

Emerging data suggest that neuroinflammation plays a critical role in the pathophysiology of MDD, particularly in its more severe and treatment-resistant forms [47]. With immune-mediated processes significantly influencing brain function, inflammation in the CNS is now understood to be a primary cause of depressive illness rather than a supporting actor [48]. This section investigates how neuroinflammatory processes, which are characterized by immunological activation, cytokine dysregulation, and metabolic imbalance, interact with brain areas involved in mood regulation. Novel therapeutic approaches that target these inflammatory pathways, such as phytochemical therapies, are becoming more popular as promising adjuncts in the treatment of depression as knowledge grows.

Neuroinflammation, driven by immune activity in the CNS, is intimately associated with depression through elevated levels of pro-inflammatory cytokines and enhanced microglial activation in the prefrontal cortex, anterior cingulate cortex, and insula [49,50,51,52,53,54]. Among the key cytokines, interleukin (IL)-1β can interfere with neurotransmitter systems and trigger more inflammatory substances, while IL-6 often increases in people with depression, affecting the normal function of the hypothalamic–pituitary–adrenal axis [55,56]. Tumor necrosis factor-α (TNF-α), known for its potent pro-inflammatory role, influences synaptic transmission and neural plasticity [57]. Interferon-gamma (IFN-γ) boosts inflammation in the brain, while IL-2, IL-8, and IL-18 create a harmful inflammatory situation [58,59]. In the prefrontal cortex, microglia adopt a pro-inflammatory phenotype, producing high levels of cytokines that may impair neuronal health [60]. When these cellular sentinels remain on high alert, they release toxic mediators that compromise neuronal functioning and hamper communication between circuits [60]. Similar inflammatory activity in the anterior cingulate cortex affects emotional processing, and overactive microglia in the insula intensify negative emotional states [61]. The hippocampus and amygdala also display maladaptive immune reactivity, undermining neuroplasticity and fueling the progression of depressive symptoms [62].

Approximately 30% of persons with MDD have substantial neuroinflammation in the CNS, which is associated with increased symptom intensity and resistance to standard treatments [47,63]. Neuroinflammation is a key factor in the pathogenesis of MDD, with activation of the NLRP3 inflammasome leading to elevated pro-inflammatory cytokines like IL-1β and IL-18, which are linked to depressive symptoms [64,65,66]. Activation of pattern recognition receptors, such as Toll-like receptor 4 (TLR4) on microglia and astrocytes, can induce NF-κB-driven cytokine production when damage or pathogens are detected [67]. Peripheral immune cells invade the CNS via a weakened blood–brain barrier, exacerbating local inflammation and neurotoxicity [68]. Chronic inflammation exacerbates oxidative and nitrosative stress, damages mitochondria, and enhances glutamate excitotoxicity through reduced astrocytic reuptake, resulting in neuronal injury [69]. Dysfunctional mitochondria produce pro-inflammatory chemicals, which promote inflammation [70]. Chronic stress-induced cortisol elevations activate microglia, whereas gut–brain axis abnormalities allow bacterial endotoxins to worsen systemic inflammation [71]. Last but not least, inflammatory kynurenine pathway activation lowers serotonin synthesis, which feeds a vicious cycle of depression [72].

Inflammatory cascades, which are characterized by sustained activation of cytokines and immune responses, are not limited to depression; they are also found in various chronic infections, neurodegenerative disorders such as multiple sclerosis, and perinatal depression, demonstrating their widespread influence across pathologies [73,74,75,76,77,78,79]. Chronic infections continue to excite immune cells, resulting in elevated amounts of pro-inflammatory cytokines, which contribute to mood and cognitive impairment [80]. Analogously, autoimmune-mediated inflammation is a feature of neurodegenerative diseases like multiple sclerosis, in which protracted immune responses harm brain tissue, aggravating symptoms of depression [75]. In prenatal depression, immunological activation triggered by hormonal and physiological changes greatly increases inflammation, affecting both maternal mood and child outcomes [81]. Furthermore, immunological dysregulation frequently interacts with metabolic changes such as neuroendocrine dysfunction, gut microbiota dysbiosis, and mitochondrial abnormalities, all of which exacerbate depressive states [82,83,84,85]. Neuroendocrine disruptions, such as cortisol imbalance due to hypothalamic–pituitary–adrenal axis dysfunction, exacerbate inflammation and depressive symptoms [86]. Gut dysbiosis promotes systemic inflammation by increasing intestinal permeability and endotoxin exposure, whereas mitochondrial dysfunction impairs cellular energy metabolism and increases oxidative stress [87]. These interconnected inflammatory and metabolic abnormalities highlight the complicated, diverse pathophysiology of depression.

Given the importance of neuroinflammation in the pathogenesis of severe and treatment-resistant depression, identifying effective therapeutic strategies that target inflammation is critical [47,88]. Notably, accumulating evidence suggests that phytochemical therapies, such as polyphenols, may provide neuroprotective advantages by inhibiting inflammatory pathways and lowering neuronal death, making them a promising adjunct method for treating MD [73,74]. Natural substances can protect neurons by modulating inflammation, inhibiting NF-κB activation, and reducing oxidative stress. Furthermore, polyphenols may repair dysregulated neurotransmission and increase mitochondrial function, addressing the basic metabolic abnormalities seen in depressed states [89]. Because neuroinflammation is so tightly linked to metabolic dysfunction, immunological dysregulation, and neuronal degeneration, therapies that target numerous inflammatory and apoptotic pathways at the same time may produce better therapeutic results [90]. Future research should emphasize investigating the entire therapeutic potential of phytochemicals and comparable molecules as supplemental treatments, which could improve outcomes for people suffering from severe, inflammation-related depression.

## 3. Oxidative Stress: A Silent Driver of Depression

Amid the complex web of MDD pathology, oxidative stress has emerged as a subtle yet powerful contributor deserving closer scrutiny [90]. Characterized by an imbalance between reactive oxygen species (ROS) and antioxidant defenses, oxidative stress quietly undermines neuronal integrity and brain function [91]. Intriguingly, mounting evidence suggests that this biochemical imbalance not only parallels depressive symptoms but may actively drive their persistence and severity [92]. From antioxidant depletion to mitochondrial dysfunction, oxidative stress orchestrates a cascade of cellular disruptions [93]. In this section, we explore the biochemical undercurrents of oxidative stress and its far-reaching implications in the development and progression of depression.

Oxidative stress occurs when ROS and other free radicals exceed the body’s capacity to neutralize them through antioxidant defenses, leading to cellular harm via the degradation of essential biomolecules such as proteins, lipids, and nucleic acids [94,95]. In individuals with depression, this imbalance is particularly pronounced due to significantly reduced levels of key antioxidants, including zinc, vitamin E, and coenzyme Q10, amplifying cellular vulnerability and intensifying neurodegenerative processes [91,96]. These antioxidant deficits facilitate oxidative damage to neuronal membranes, impair mitochondrial function, and disrupt neurotransmission, further exacerbating depressive symptoms [95]. Zinc deficiency, for example, diminishes neuroplasticity and impairs glutamatergic signaling, whereas insufficient vitamin E reduces neuronal protection against lipid peroxidation [97]. Likewise, low levels of coenzyme Q10 impair mitochondrial respiration, increasing ROS production and energy deficits in neurons [98]. Collectively, this heightened oxidative state promotes neuronal apoptosis and inflammation, reinforcing a destructive cycle that sustains and deepens depressive pathology [89]. Thus, addressing oxidative imbalances through antioxidant supplementation or dietary interventions could potentially mitigate cellular damage and improve therapeutic outcomes in MDD.

Alterations in tryptophan metabolism significantly contribute to oxidative stress in MDD, primarily by shifting this amino acid toward the kynurenine pathway rather than serotonin synthesis. When inflammation activates enzymes such as indoleamine 2,3-dioxygenase (IDO), tryptophan is increasingly converted into kynurenine metabolites, which not only reduce serotonin availability but also generate neurotoxic byproducts like quinolinic acid [19,99,100]. These toxic intermediates promote neuronal damage through enhanced generation of ROS, exacerbating oxidative stress and accelerating cellular injury within vulnerable brain regions [91]. Furthermore, reduced tryptophan availability itself can intensify the formation of free radicals, damaging essential biomolecules including deoxyribonucleic acid (DNA), lipids, and proteins [93]. This cascade creates a destructive cycle of oxidative damage, inflammation, and impaired neurotransmission, significantly correlating with increased severity of depressive symptoms [91]. The resulting neuronal dysfunction, loss of synaptic plasticity, and impaired mitochondrial performance contribute to the pathophysiology of depression [101]. Therapeutic interventions targeting the kynurenine pathway or enhancing tryptophan availability thus hold considerable potential for reducing oxidative damage and alleviating depressive symptoms.

Mitochondrial dysfunction—including structural alterations and disrupted energy metabolism—significantly escalates ROS production, directly promoting neuroinflammation and cognitive decline in depressive disorders [102,103,104,105]. Accumulation of dysfunctional mitochondria further exacerbates neuronal damage, perpetuating chronic inflammatory cycles within the brain [106,107]. Specifically, impaired mitophagy hampers the removal of damaged mitochondria, allowing toxic debris to accumulate and activate inflammatory complexes, notably the NLRP3 inflammasome, leading to increased pro-inflammatory cytokine production [108]. Disrupted mitochondrial calcium buffering elevates intracellular calcium, triggering oxidative damage, microglial activation, and amplified inflammatory signaling [109]. Moreover, mitochondrial energy deficits dysregulate AMP-activated protein kinase (AMPK) signaling, causing aberrant mammalian target of rapamycin (mTOR) activation that sustains inflammatory responses [110]. Increased ROS production triggers transcription factors such as NF-κB, leading to ongoing release of inflammatory cytokines (IL-6 and TNF-α) [111]. Reduced mitochondrial health also lowers NAD^+^ levels, which weakens protective sirtuin signaling, while imbalanced iron levels encourage ferroptosis, worsening oxidative damage [90,112]. Consequently, impaired neurogenesis and reduced synaptic plasticity intensify cognitive deficits, reinforcing a destructive feedback loop of mitochondrial-driven inflammation in depression [107].

Obesity-induced inflammation significantly heightens oxidative stress by increasing adipokine secretion—such as IL-6, IL-1, and tumor necrosis factor-alpha—while simultaneously impairing insulin regulation, thus elevating risks for type 2 diabetes and depression [113,114,115]. Chronic obesity also fosters leptin resistance, amplifying microglial activation and further promoting inflammatory cascades within the brain [116]. Moreover, lifestyle factors, particularly high-fat diets and sedentary behaviors, escalate ROS production through enhanced mitochondrial β-oxidation and activation of nuclear factor kappa B (NF-κB) signaling, reinforcing inflammation and neuronal stress pathways [117,118,119,120,121]. Dietary saturated fats directly stimulate inflammasome complexes like NLRP3, causing excessive IL-1β release and perpetuating neuroinflammation [122]. Additionally, obesity-related hyperglycemia accelerates formation of advanced glycation end-products (AGEs), activating inflammatory responses via AGE receptors on microglia [123]. High-fat diets exacerbate gut microbiota disruption, increasing endotoxin leakage into circulation and systemic inflammation while simultaneously lowering BDNF levels, impairing neuronal resilience [124]. These interconnected metabolic, inflammatory, and behavioral mechanisms underscore the critical role of dietary antioxidants and regular physical exercise in mitigating inflammation-induced depressive pathology.

Oxidative stress stands as a potent yet often overlooked force driving the progression and persistence of MDD [95]. Rather than being a mere consequence of other pathological changes, it plays an active role in linking mitochondrial dysfunction, immune activation, metabolic disturbances, and adverse lifestyle factors [125]. Through sustained production of ROS and insufficient antioxidant defenses, oxidative stress damages cellular structures, fuels neuroinflammation, and disrupts key neural circuits involved in mood regulation [95]. Its impact extends from molecular disruption to large-scale cognitive decline and emotional dysregulation [126]. Addressing this underlying imbalance—via antioxidant therapies, metabolic support, and targeted lifestyle changes—offers a promising direction for enhancing treatment response and improving long-term outcomes in individuals with depression.

## 4. Depression’s Mitochondrial Roots and Repair Strategies

Far beyond their role as mere energy suppliers, mitochondria are now recognized as pivotal regulators of brain health and emotional resilience [127]. In the context of MDD, mitochondrial dysfunction has garnered growing attention as a hidden yet potent driver of neurobiological disruption [112]. From impaired energy metabolism to excessive ROS production, mitochondrial abnormalities appear to fuel the progression of depressive symptoms [89]. This section delves into the emerging landscape of mitochondrial pathology in depression—alongside innovative repair strategies—highlighting a compelling shift in how we understand and potentially treat this multifaceted disorder.

Mitochondria also play a pivotal role in regulating neuroinflammation, positioning them as key contributors to both the onset and persistence of MDD [85]. In individuals with MDD, mitochondrial abnormalities—such as structural degradation, disrupted mitochondrial DNA (mtDNA), and impaired electron transport chain (ETC) function—lead to excessive production of ROS, destabilizing neurotransmitter systems and exacerbating depressive symptoms [103,128,129,130]. These disturbances are intensified by additional mitochondrial impairments [131]. Oxidative damage to mtDNA impairs the synthesis of ETC components, while dysregulated biogenesis reduces the formation of healthy mitochondria [132]. Imbalances in fusion and fission dynamics further fragment the mitochondrial network, diminishing metabolic capacity and increasing susceptibility to cell death [133]. Chronic stress-related calcium overload, inhibition of ETC complexes, and glucocorticoid-induced suppression of mitochondrial maintenance genes all contribute to dysfunction [134]. Moreover, a disrupted NAD^+^/NADH ratio and persistent exposure to pro-inflammatory cytokines impair mitochondrial respiration, DNA repair, and anti-inflammatory signaling [135]. Together, these interconnected mechanisms establish a vicious cycle of mitochondrial breakdown, oxidative stress, and neuroinflammation that drives the neurobiological deterioration seen in MDD.

While psychosocial stressors undoubtedly contribute to the onset of MDD, a growing body of evidence highlights the role of biochemical markers—particularly those tied to mitochondrial health—in its pathophysiology [102]. One such marker, methylmalonic acid (MMA), is elevated in cases of vitamin B12 deficiency and has been shown to impair succinate dehydrogenase activity, thereby increasing ROS production and promoting neuronal injury [136,137,138,139,140]. Additional biomarkers further implicate mitochondrial dysfunction in MDD. Elevated homocysteine, another consequence of B-vitamin deficiency, induces oxidative stress and compromises mitochondrial DNA repair [141]. Increased brain lactate and pyruvate levels suggest a shift from oxidative phosphorylation to inefficient anaerobic metabolism [142]. Accumulation of acylcarnitines reflects disrupted fatty acid oxidation, while elevated ammonia impairs mitochondrial respiration and contributes to neurotoxicity [143]. Depletion of glutathione (GSH), the mitochondria’s frontline antioxidant, leaves cells vulnerable to ROS, and heightened levels of 8-hydroxy-2′-deoxyguanosine (8-OHdG) indicate mtDNA damage [144]. Deficiencies in coenzyme Q10 and imbalances in the NAD^+^/NADH ratio further hinder ATP production and stress resilience [145]. Together, these markers underscore the biochemical complexity and mitochondrial vulnerability underlying depressive disorders. These observations resonate with broader neurodegenerative research frameworks, which emphasize the integration of imaging biomarkers, inflammatory profiles, and metabolic signatures to characterize disease subtypes and guide personalized intervention strategies [146].

Recent studies underscore several promising therapeutic strategies aimed at correcting mitochondrial dysfunction in MDD [147]. Among them, mitochondrial transplantation—either by replacing damaged mitochondria or introducing healthy mitochondrial DNA and related proteins—has emerged as a novel approach to restoring cellular energy balance and reversing neuronal damage [148]. Similarly, inhibiting the renin–angiotensin system with angiotensin receptor blockers has shown potential in reducing neuroinflammation and oxidative stress, both key drivers of depressive pathology [149,150]. Beyond these, mitochondria-targeted antioxidants like MitoQ and SkQ1 directly neutralize ROS within mitochondria, improving membrane potential and behavioral outcomes [151]. Supplementation with NAD^+^ precursors (e.g., nicotinamide riboside) enhances mitochondrial biogenesis and DNA repair via sirtuin activation [152]. Coenzyme Q10 boosts ATP production, while PGC-1α activators such as resveratrol and AMPK activators promote mitochondrial renewal [153,154]. Other emerging strategies include inhibition of the mitochondrial permeability transition pore (mPTP), ketogenic diets to optimize mitochondrial fuel use, sirtuin activators to dampen inflammation, and L-carnitine derivatives that support fatty acid oxidation [155,156,157,158]. Gene therapies targeting mtDNA mutations offer future avenues for correcting mitochondrial defects at their source [159].

Ultimately, restoring mitochondrial health offers a compelling path forward in the treatment of MDD. By integrating phytochemicals and other multimodal strategies—ranging from targeted antioxidants to metabolic and genetic interventions—it may be possible to reduce oxidative damage, enhance energy metabolism, and reinforce neuronal resilience [102]. These approaches hold promise not only for symptom relief but also for addressing the cellular dysfunctions at the core of depression’s pathology. Importantly, mitochondrial fragility does not occur in isolation. Caloric excess, insulin resistance, and adipose-derived cytokines impose a chronic oxidative–inflammatory load that further erodes mitochondrial ATP output and calcium buffering in limbic neurons. In turn, energy-starved mitochondria dysregulate appetite and glucose homeostasis via hypothalamic circuits, creating a vicious cycle in which metabolic inflammation and mitochondrial dysfunction co-amplify depressive pathology. This bidirectional loop sets the stage for examining obesity-linked inflammation in greater detail.

## 5. Nutrition, Inflammation, and Depressive Spirals

While nutrition and lifestyle factors clearly shape metabolic risk and modulate inflammation relevant to MDD, a detailed discussion lies beyond the core focus of this review. We briefly highlight key findings to underscore how diet quality, physical activity, and sleep patterns influence neuroimmune health. These modifiable behaviors may complement pharmacological and phytochemical strategies, but a full exploration of their therapeutic applications warrants separate, dedicated analysis. Nutrition lies at the crossroads of physical and mental health, yet its role in MDD is often underestimated [160]. Increasingly, research points to a vicious cycle in which poor dietary habits not only reflect depressive states but actively intensify them through metabolic disruption and chronic inflammation [161]. As nutrient deficiencies and unhealthy food choices fuel neurobiological stress, depressive symptoms may deepen, further eroding motivation for self-care [162]. This section explores how diet, lifestyle, and inflammation intertwine in self-perpetuating spirals—offering both a cautionary tale and a hopeful lens on how strategic nutritional interventions may help break the cycle.

Recent lines of evidence highlight that dietary behaviors, modulated by psychological factors, significantly shape obesity risk and depressive symptoms by influencing nutrient availability and metabolic function [163,164,165]. In particular, inadequate consumption of vitamin B12, zinc, magnesium, folic acid, and vitamin B6 has been associated with elevated susceptibility to MDD [15]. Moreover, diets dominated by high-fat convenience foods often coincide with lower intake of fruits, vegetables, lean proteins, and whole grains, fueling systemic inflammation and oxidative stress linked to depression pathogenesis [166,167,168]. These findings underscore the multifaceted interplay among dietary patterns, nutritional deficiencies, and mood disorders, suggesting that suboptimal eating habits may potentiate neurobiological pathways underlying depression [169]. By integrating these insights into clinical practice, researchers and clinicians can more effectively target modifiable lifestyle factors in conjunction with pharmacotherapy or psychotherapy [170]. Ultimately, developing evidence-based nutritional interventions, particularly those emphasizing nutrient-rich whole foods, could mitigate depressive symptom severity while reducing broader metabolic risk [171]. This comprehensive approach holds promise for reducing disease burden and improving patient outcomes in MDD.

Obesity is increasingly recognized as a critical comorbidity in depression, given that excess adiposity promotes a pro-inflammatory milieu and metabolic dysregulation, potentially exacerbating psychiatric symptomatology [83,84]. In parallel, emerging data indicate that individuals with severe depressive disorders often exhibit behaviors that intensify these physiological burdens, such as smoking, physical inactivity, and insufficient sleep [172,173]. These lifestyle factors further elevate systemic inflammation and oxidative stress, reinforcing a feedback loop that intensifies both mood disturbances and metabolic risk [47]. Notably, recent studies underscore that even modest reductions in body mass index or targeted interventions to improve sleep quality can substantially attenuate neuroinflammatory processes linked to depression [174]. By highlighting these interdependent mechanisms, the present review affirms the need for comprehensive management approaches that address not only psychological distress but also modifiable health behaviors [170]. Tailored interventions that incorporate nutritional counseling, physical activity regimens, and sleep hygiene may thus hold therapeutic promise, mitigating the dual burden of metabolic and mental health complications [175,176]. Advancing this integrative perspective could ultimately refine treatment algorithms and inform future research directions in translational psychiatric care.

Emerging data consistently underscore the potential for lifestyle modifications to alleviate mood-related symptomatology and address comorbid metabolic disorders in individuals with depression [170]. Notably, adopting healthier diets characterized by nutrient-dense foods, minimized refined sugars, and balanced macronutrient intake appears to diminish systemic inflammation and improve metabolic regulation [177]. When combined with regular physical activity, such dietary patterns may enhance neuroplasticity through increased neurotrophic factor release, supporting neuronal resilience [178]. Stress management techniques, including mindfulness-based practices and cognitive–behavioral strategies, further mitigate hypothalamic–pituitary–adrenal axis dysregulation, thereby moderating pro-inflammatory responses and bolstering mental health [165,179,180]. Although these findings remain preliminary, they collectively suggest that targeted lifestyle programs can serve as essential components of comprehensive treatment plans [181]. Moreover, recent randomized controlled trials affirm that multifaceted approaches integrating nutritional counseling, behavioral interventions, and exercise regimens yield meaningful improvements in depressive symptoms and metabolic risk profiles [182]. This perspective highlights the need to broaden therapeutic frameworks beyond pharmacological paradigms to address the complex interplay among diet, behavior, and psychological well-being in depression [183].

Collectively, these findings illuminate how poor dietary habits can instigate a vicious cycle in which chronic inflammation exacerbates depressive pathology, further eroding motivation for healthy behavior [161]. By perpetuating neurobiological stress, this spiral intensifies symptom severity and increases metabolic risk, reinforcing suboptimal eating patterns [161]. Adopting balanced, nutrient-rich diets, regular physical activity, and evidence-based stress management may disrupt this harmful feedback loop, thereby enhancing neurotransmitter balance, mitigating systemic inflammation, and restoring metabolic homeostasis [170]. Although rigorous trials remain necessary to pinpoint optimal protocols, strategic nutritional interventions hold considerable promise for curtailing the depressive spiral and advancing patient-centered treatment paradigms in MDD [184].

## 6. Rethinking Depression Care: Drugs and Natural Options

As our understanding of MDD deepens, so too does the landscape of its treatment—now evolving beyond traditional pharmacotherapy toward more integrative, personalized care [185]. While established medications like SSRIs and SNRIs remain central, their side-effect profiles and limitations prompt a reexamination of what comprehensive care can look like [186]. Intriguingly, nature itself may offer untapped solutions [187]. From time-honored botanicals to novel phytochemicals, natural compounds are gaining recognition as adjunctive or alternative treatments with promising efficacy and improved tolerability [188]. This section explores the growing synergy between conventional drugs and natural options in the quest for more holistic depression care.

Pharmacological and non-pharmacological therapies for MDD are increasingly being delivered through multidisciplinary care teams, reflecting a shift toward more holistic, patient-centered treatment models [189,190]. These teams typically include psychiatrists—who oversee diagnosis, medication management, and complex case coordination—and clinical psychologists or psychotherapists, who deliver evidence-based therapies such as CBT, IPT, and mindfulness-based interventions [191,192]. Nutritionists or dietitians also play a key role by addressing nutritional deficiencies and promoting anti-inflammatory dietary patterns that support mitochondrial and mental health [191,193]. Exercise physiologists or physical therapists prescribe tailored physical activity regimens to reduce depressive symptoms and improve neurobiological function [191,192]. Primary care physicians manage medical comorbidities that often worsen mood disorders, while pharmacists ensure medication safety and adherence [194]. Complementary and integrative health practitioners may introduce acupuncture, yoga, or nutraceutical support to reduce oxidative stress [195]. Social workers and case managers address psychosocial barriers and improve continuity of care, and peer support specialists offer lived-experience insights that build trust and engagement [196]. This interdisciplinary approach is especially effective in treatment-resistant or complex cases, offering a comprehensive strategy that integrates biological, psychological, and social dimensions of depression [197,198,199].

Among pharmacological options for MDD, selective serotonin reuptake inhibitors (SSRIs) remain the preferred first-line treatment due to their efficacy and relative tolerability. However, alternative classes such as serotonin–norepinephrine reuptake inhibitors (SNRIs), TCAs, monoamine oxidase inhibitors (MAOIs), and N-methyl-D-aspartate (NMDA) antagonists provide additional therapeutic pathways, especially in treatment-resistant cases [10,200,201,202]. Despite their benefits, these agents are associated with a range of adverse effects [203]. SSRIs can cause gastrointestinal disturbances, insomnia, sexual dysfunction, and in some cases, QTc prolongation—particularly with citalopram [204]. SNRIs like venlafaxine and duloxetine may induce hypertension, hepatotoxicity, and withdrawal symptoms [205]. TCAs carry anticholinergic burdens (e.g., dry mouth, constipation) and significant cardiovascular risks, including arrhythmias [206]. MAOIs, though effective, are limited by dietary restrictions and the risk of hypertensive crises or serotonin syndrome [207]. Atypical antidepressants such as bupropion and mirtazapine offer varied mechanisms but introduce risks like insomnia, weight gain, or seizure potential [208]. Novel agents, including NMDA antagonists like esketamine, present dissociative and cardiovascular side effects [209]. Given these diverse profiles, careful, individualized treatment planning is essential [208,210,211,212,213]. The limitations of conventional antidepressants, such as the risk of hyponatremia in older adults and individuals taking diuretics, underscore the need for safer and more tolerable treatment options [214,215].

These safety concerns have prompted increasing interest in alternative and complementary strategies that can enhance efficacy while reducing adverse effects [216]. Herbal medicines are gaining recognition as potential adjuncts in the treatment of MDD [217]. These natural compounds may exert neuroprotective, anti-inflammatory, and mood-regulating effects while offering better tolerability and fewer side effects compared to standard medications [218,219,220]. By improving safety profiles and supporting long-term adherence, herbal therapies may help address some of the limitations associated with conventional pharmacological approaches [221]. Their integration into clinical practice could also support a more holistic and individualized model of care [222]. As research continues to explore these compounds’ mechanisms and clinical potential, combining pharmacological and natural interventions may represent a promising path toward more comprehensive and patient-centered depression management.

## 7. Plant-Based Therapies: Natural Allies Against Depression

As science turns its gaze toward nature’s pharmacopoeia, plant-based therapies are emerging as exciting frontiers in the treatment of MDD [223]. Rich in bioactive compounds, plants offer a diverse array of phytochemicals capable of modulating the same neurobiological pathways targeted by conventional antidepressants—often with fewer side effects [224]. From polyphenols and flavonoids to carotenoids and alkaloids, these natural agents show potential to combat oxidative stress, inflammation, and neurodegeneration [225]. This section delves into the growing body of evidence supporting phytotherapies as integrative, multifaceted allies in the fight against depression—opening the door to greener, gentler innovations in mental health care.

Phytochemicals, as bioactive compounds naturally derived from plants, have attracted increasing interest for their ability to modulate key neurobiological pathways involved in MDD [37,226]. Among them, polyphenols are particularly noteworthy for their ability to cross the blood–brain barrier and exert neuroprotective, antioxidant, and anti-inflammatory effects, thereby attenuating cytokine activity and reducing neuronal apoptosis [74,227]. Within this class, flavonoids such as luteolin effectively scavenge free radicals and suppress neuroinflammation [227,228,229], while carotenoids offer similar antidepressant benefits through antioxidant and anti-inflammatory mechanisms [230,231,232,233]. These effects align with the growing evidence that enhancing BDNF signaling improves synaptic plasticity and emotional regulation. Compounds like curcumin and resveratrol have demonstrated the ability to upregulate BDNF in animal models, contributing to mood stabilization and resilience [234,235,236]. Additional polyphenols—including quercetin, EGCG, fisetin, apigenin, and baicalein—further reinforce these effects by modulating inflammatory signaling, reducing oxidative stress, and promoting mitochondrial function [237,238,239,240]. Collectively, these natural agents represent promising adjunctive therapies for improving clinical outcomes in MDD through multimodal neuroprotective actions.

Additional phytochemicals found in medicinal plants such as *Panax ginseng*, *Mitragyna speciosa*, *Astragalus membranaceus*, and species within the *Acorus* genus have shown antidepressant-like effects primarily through their anti-inflammatory and antioxidant properties [32,34,241,242,243]. These effects are echoed in other plant-derived compounds that act on convergent molecular pathways [130]. Withanolides from *Withania somnifera* modulate the HPA axis, reduce pro-inflammatory cytokines like IL-6 and TNF-α, and elevate BDNF levels, enhancing stress resilience [244]. Hericenones and erinacines from *Hericium erinaceus* stimulate nerve growth factor synthesis, reduce oxidative stress, and improve monoaminergic balance [245]. Ginsenosides from *Panax ginseng* and bacopasides from *Bacopa monnieri* both boost mitochondrial efficiency, suppress neuroinflammation, and promote neuroplasticity [130,246]. Other promising agents include honokiol from *Magnolia officinalis*, paeoniflorin from *Paeonia lactiflora*, and berberine from *Berberis* species, all of which influence oxidative, inflammatory, and neurotransmitter systems [247,248,249]. Rhynchophylline and salidroside further contribute to this phytochemical network by protecting neurons and regulating mood circuits [250]. Collectively, these compounds offer diverse mechanisms for mitigating the multifaceted pathways underlying mood disorders by reducing inflammation, alleviating oxidative stress, fine-tuning neurotransmitter balance, and fostering neuroplasticity, thereby highlighting their integrative therapeutic potential.

**Figure 2:**
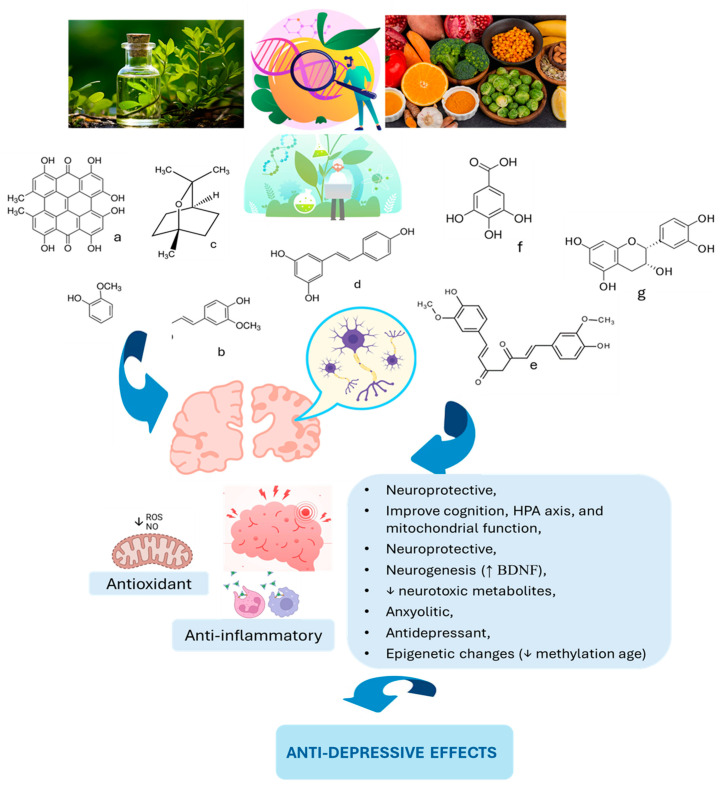
Phytochemicals and the role in brain health and reduction in depression. HPA: hypothalamic–pituitary–adrenal; BDNF: brain-derived neurotrophic factor; a: hypericin; b: coumarin; c: eucalyptol; d: resveratrol; e: curcumin; f: gallic acid; g: epicatechin. ↑: increase; ↓: decrease.

Although plant-based interventions often exhibit fewer side effects than conventional antidepressants, their clinical application still requires deeper investigation to refine optimal dosing, clarify underlying mechanisms, and ensure long-term safety [251,252]. Nonetheless, mounting evidence supports the potential of phytotherapeutics as effective, integrative strategies for addressing the complex and multifactorial nature of MDD [1]. By targeting neuroinflammation, oxidative stress, mitochondrial dysfunction, and neurotransmitter imbalances, these compounds offer a multi-pronged approach to mood regulation [253]. Their ability to enhance neurotrophic factors such as BDNF further strengthens their therapeutic value [254]. As the field of psychoneuropharmacology evolves, incorporating plant-derived compounds into treatment frameworks may improve outcomes for patients who are unresponsive or intolerant to traditional therapies [255]. Collectively, these findings highlight the critical need for continued clinical research on phytomedicines in mood disorder management [256]. Figure 2 illustrates select phytochemicals and their modulatory effects on brain metabolism and neural function, underscoring their relevance as natural allies in depression care.

## 8. From Curcumin to Cocoa: Plant-Based Mood Solutions

The following interventional studies reveal a promising though heterogeneous body of evidence regarding the therapeutic potential of phytocompounds in MDD. These trials examined a wide range of phytochemicals, including curcumin, quercetin, polyphenols, anthocyanins, isoflavones, proanthocyanidins, and flavonoid-rich extracts, often administered in monotherapy or adjunctive formats. Although sample sizes, duration, and design quality varied, most studies used validated depression scales and biochemical endpoints, supporting a neurobiological rationale for clinical translation.

In a well-structured randomized controlled trial, Soltani et al. [25] evaluated the impact of nanocurcumin supplementation in individuals with coronary slow flow phenomenon (CSFP). The intervention led to improvements in depressive symptoms, physical and psychological health-related quality of life, and cardiometabolic parameters, with biochemical assays confirming anti-inflammatory and antioxidant effects [257]. In another study examining quercetin effects in patients who have experienced myocardial infarction, the authors did failed to not find significant antidepressant effects [258].

Methodological strengths such as stratified randomization and biomarker analysis were evident in the study by Hajiluian et al. [34], which demonstrated reduced depressive symptoms in individuals with multiple sclerosis following polyphenol-rich interventions. However, its narrow population limits generalizability to broader MDD cohorts [259]. In a similarly well-structured clinical trial, Choi et al. [27] examined the effects of flavonoid-enriched orange juice in healthy adults. Improvements in depressive symptoms were paralleled by increased serum BDNF and reduced zonulin levels, indicating neuromodulatory and gut–brain axis effects. Despite robust biochemical correlates, the sample size of 40 reduced statistical power [27].

Maeda-Yamamoto et al. [35] assessed the impact of anthocyanin-rich *Solanum tuberosum* L. on stress and mesenchymal stem cell proliferation. Although qualitative outcomes were positive, the trial was limited by its very small sample (*n* = 15) and short duration (8 weeks), which precluded long-term conclusions. Similarly, Barfoot et al. [36] conducted a randomized study during the COVID-19 pandemic, which introduced significant confounding related to global psychological stress. While randomization via software strengthened validity, high dropout rates impaired data reliability.

Parilli-Moser et al. [37] explored the effects of botanical cognitive enhancers on depressive symptoms. Although standardized cognitive assessments were used, low group sizes and a lack of blinding limited interpretive strength and COVID-19-related disruptions likely impacted outcome validity. In contrast, the RISTOMED study by Bourdel-Marchasson et al. [38] assessed a dietary protocol rich in antioxidants and polyphenols. After 2 months, reductions in depressive symptoms were observed, but these were found to be independent of inflammatory biomarker changes, suggesting mechanisms beyond systemic inflammation.

Kontogianni et al. [260] investigated high versus low polyphenol diets in the PPhIT trial, noting improved psychological well-being in the high-polyphenol group using standardized lifestyle and mood assessments. Park, Choi, and Lee [260] similarly conducted a placebo-controlled flavonoid study using orange juice and observed improvements in depression alongside increased serum serotonin, BDNF, and reduced CRP—though the small sample again limits external validity.

Smetanka et al. [260] focused on the potential for pycnogenol to mitigate SSRI-induced sexual dysfunction in patients taking escitalopram. However, the open-label design and simultaneous pharmacologic interventions created confounding that weakens causal inference. In the curcumin trial by Kanchanatawan et al. [40], methodological strengths included matched placebo capsules and double-blinding, although prior treatments continued during the trial, potentially masking true curcumin effects.

The study by Esmaily et al. [41] observed only marginal reductions in anxiety and depressive symptoms in response to saffron extract, with effects limited to a single outcome measure—suggesting potential underpowering or scale insensitivity. In a postmenopausal population, Terauchi et al. [33] demonstrated that grape seed proanthocyanidins reduced depressive symptoms in a dose-dependent manner, using validated questionnaires. However, the short duration (8 weeks) and modest sample size limit generalizability.

Equol and resveratrol were evaluated in a longitudinal 12-week study on menopausal women aged 50–55, where supplementation improved mood as assessed by the Hamilton depression rating scale (HAM-D) [261]. Hirose et al. [262] explored low-dose isoflavone aglycone for postmenopausal symptoms, including depression and anxiety, using HADS and AIS. Despite positive outcomes, lack of adverse event documentation and small sample size limit clinical confidence.

Cognitive-affective benefits of blueberry-derived flavonoids were tested in a crossover design by Khalid et al. [262], involving both children and young adults. Stratified analysis revealed reductions in depressogenic cognitive patterns two hours after consumption, supporting the acute neuromodulatory potential of these compounds.

Lastly, Sathyapalan et al. [262] evaluated chocolate rich in cocoa liquor and polyphenols versus low-polyphenol control chocolate in patients with chronic fatigue syndrome. Participants in the high-cocoa group reported improvements in depressive and fatigue symptoms, supporting flavonoid efficacy in neuropsychological syndromes. Pase et al. [11] reported mood-enhancing effects of cocoa polyphenols but no cognitive improvements after 30 days of supplementation.

Overall, these reviewed studies underscore the emerging promise of phytocompounds as adjunctive or standalone interventions for MDD (Table 1). While most trials reported clinically meaningful reductions in depressive symptoms, variability in methodological rigor, sample size, and treatment duration limited the generalizability of these findings. Notably, studies incorporating double-blind, placebo-controlled designs and objective biomarkers (e.g., inflammatory mediators neurotrophic factors) provided stronger evidence for neuromodulatory and neuroprotective mechanisms. Nevertheless, several investigations reported inconclusive or modest effects, indicating the necessity of improved trial designs and larger, more diverse samples. Given the heterogeneous nature of depression, a multi-targeted approach—such as that offered by phytochemicals—holds particular relevance. Future research efforts should focus on optimizing dosage regimens, exploring synergistic effects with standard antidepressants, and elucidating long-term safety profiles to solidify phytocompounds’ place in evidence-based psychiatric care.

## 9. Discussion

MDD remains a complex, multifaceted psychiatric condition that resists simple explanations or treatments [275]. The primary goal of this review was to explore how phytochemicals—naturally occurring plant-derived compounds—may offer a biologically plausible and clinically relevant approach to modulating the intricate neurobiological disruptions observed in MDD [1]. Central to the disorder’s pathophysiology are sustained neuroinflammation, oxidative stress, mitochondrial dysfunction, and metabolic disturbances, which interact to compromise neuronal integrity, synaptic plasticity, and emotional regulation [276]. While monoaminergic deficits have long dominated depression theories, current evidence indicates these downstream effects may be fueled by deeper cellular and immune dysregulation [277]. Phytocompounds such as polyphenols, flavonoids, and alkaloids appear to influence these upstream processes by attenuating pro-inflammatory cytokines, scavenging ROS, restoring mitochondrial dynamics, and modulating neurotrophic signaling [136]. In doing so, they challenge conventional paradigms and suggest a more integrative neuropsychiatric model—one that views depression through the lens of systemic inflammation and neuroenergetic compromise rather than solely neurotransmitter depletion [278].

A central finding of this review is the growing body of evidence supporting the antidepressant potential of phytochemicals, particularly polyphenols, flavonoids, and alkaloid-rich plant extracts. Compounds such as curcumin, resveratrol, quercetin, and luteolin consistently demonstrate antidepressant-like effects across preclinical models and emerging clinical studies. These benefits are mediated through a combination of neuroprotective, anti-inflammatory, and antioxidant mechanisms. Notably, curcumin has been shown to downregulate pro-inflammatory cytokines (e.g., IL-1β and TNF-α), reduce lipid peroxidation, and enhance BDNF expression—key pathways implicated in MDD. Similarly, flavonoids such as quercetin and luteolin attenuate microglial activation, restore mitochondrial function, and improve synaptic plasticity. These multi-target effects distinguish phytochemicals from traditional antidepressants, which primarily modulate monoamine reuptake and often require several weeks to achieve symptom relief. Moreover, phytochemicals tend to have more favorable safety profiles, presenting a lower risk of side effects such as sexual dysfunction, weight gain, or hyponatremia. For individuals with treatment-resistant depression or intolerance to conventional therapies, these natural agents may offer a promising adjunct or alternative approach. Their ability to modulate upstream drivers of MDD rather than solely downstream neurotransmitter imbalances reflects a potentially paradigm-shifting advance in how we conceptualize and manage depression.

The therapeutic potential of phytochemicals in MDD is rooted in their ability to engage a broad array of molecular targets implicated in the neurobiology of depression [279]. One of the most prominent mechanisms is the upregulation of BDNF, a key molecule in synaptic plasticity, neurogenesis, and neuronal survival [226]. Phytocompounds such as curcumin, resveratrol, and apigenin have been shown to elevate BDNF expression in preclinical models, countering the synaptic deficits commonly observed in depression [280]. Another critical mechanism involves the inhibition of the NLRP3 inflammasome, a key driver of neuroinflammation [276]. Polyphenols like quercetin and baicalein suppress this pathway, leading to decreased release of pro-inflammatory cytokines such as IL-1β and IL-18 [281]. These compounds also reduce oxidative stress by enhancing endogenous antioxidant defenses (e.g., upregulation of Nrf2 and glutathione pathways), protecting neurons from ROS-induced apoptosis [282]. Importantly, many of these phytochemicals—especially flavonoids and terpenoids—demonstrate the capacity to cross the blood–brain barrier due to their lipophilic structures and low molecular weight [283]. Once in the CNS, they can stabilize mitochondrial function, modulate calcium homeostasis, and maintain membrane potential, all of which are disrupted in MDD [284]. These mechanistic actions align closely with contemporary neurobiological models of depression, which emphasize the roles of neuroinflammation, mitochondrial dysfunction, and impaired neuroplasticity over simplistic monoamine depletion theories [89]. By targeting upstream cellular stressors and restoring homeostatic balance across neuroimmune and neurometabolic pathways, phytochemicals represent a compelling therapeutic modality that resonates with the current shift toward systems-based approaches in psychiatric research. Complementing this systems-level approach, recent insights into quinoline-based drug design suggest that subtle structural modifications, such as halogenation or esterification, can meaningfully modulate excitotoxicity and immunoactivity signaling relevant to depression pathophysiology [285].

The incorporation of phytochemicals into clinical treatment strategies for MDD presents several promising implications for practice [1]. Unlike conventional antidepressants, which often carry a high burden of side effects—such as sexual dysfunction, weight gain, insomnia, or gastrointestinal disturbances—phytochemicals typically exhibit favorable safety and tolerability profiles [256]. This reduced side-effect burden may significantly enhance patient adherence, particularly among individuals who are medication-sensitive, elderly, or managing comorbid conditions [224]. Furthermore, many phytocompounds, such as curcumin, resveratrol, and quercetin, are available in standardized formulations and have shown efficacy when used alongside standard antidepressant regimens, supporting their role as adjunctive therapies [286]. Their natural origin also makes them appealing to patients seeking holistic or integrative approaches to mental health [287]. To maximize the benefits of phytochemical interventions, multidisciplinary care teams—comprising psychiatrists, psychologists, nutritionists, pharmacists, and exercise specialists—are essential [288]. These teams can tailor phytochemical strategies to the individual’s biological and psychosocial profile, monitor for herb–drug interactions, and integrate them into broader lifestyle and behavioral interventions [289]. This integrative model not only broadens therapeutic options but aligns with growing interest in personalized medicine, where treatment is adapted to the individual’s unique physiological and lifestyle context [290].

Despite encouraging findings, several challenges and limitations temper the immediate clinical translation of phytochemicals for depression [252]. A primary concern lies in the methodological variability across studies [256]. Many preclinical and clinical trials differ widely in terms of dosage, treatment duration, and formulation, making it difficult to establish standardized protocols [1]. Sample sizes are often small, with limited representation across age groups, genders, and ethnic backgrounds, reducing the generalizability of results [291]. Moreover, key pharmacokinetic parameters—such as bioavailability, half-life, and tissue distribution—remain poorly characterized for many phytochemicals [292]. This complicates efforts to optimize dosage and timing for maximum therapeutic effect [293]. Additionally, the long-term safety of chronic phytochemical use is not well understood, particularly when used in combination with conventional antidepressants, raising concerns about potential herb–drug interactions [294]. These knowledge gaps highlight the need for rigorously designed, large-scale randomized, controlled trials that incorporate standardized extract preparations, dose-ranging studies, and biomarker-based outcome measures [295]. Future research should also emphasize population diversity and assess pharmacogenomic factors that may influence individual responses [296]. Addressing these limitations is essential for moving phytochemicals from promising adjuncts to reliable, evidence-based components of depression treatment protocols.

Future research on phytochemicals in the treatment of MDD should prioritize rigorously designed clinical trials and translational studies that bridge the gap between laboratory findings and real-world clinical application [279]. Large-scale, placebo-controlled trials are essential to validate the antidepressant efficacy of individual phytochemicals, establish optimal dosing strategies, and assess long-term safety [297]. In addition to studying single compounds, future investigations should explore the therapeutic potential of phytochemical combinations [252]. Many plant-derived compounds act on overlapping molecular targets, and their synergistic effects on neuroinflammation, oxidative stress, and mitochondrial dysfunction may offer enhanced efficacy compared to isolated agents [298]. Combinatorial approaches could mirror the polypharmacological nature of depression, targeting its multifactorial pathophysiology more effectively [225]. Moreover, research should move toward precision medicine models that account for individual differences in genetic makeup, metabolic profiles, gut microbiota composition, and inflammatory status [299]. Stratifying participants based on these biomarkers could identify responders and non-responders to specific phytochemicals, allowing for more personalized treatment [300]. Integrating omics technologies—such as metabolomics, transcriptomics, and pharmacogenomics—into trial designs will enhance mechanistic understanding and treatment customization [301]. Finally, future studies should include robust secondary endpoints that assess cognitive function, quality of life, and functional recovery, not just symptom reduction [199]. By embracing these strategies, the field can move toward the development of targeted, effective, and biologically grounded phytochemical interventions that expand the therapeutic landscape for depression.

Effective management of MDD increasingly requires a multidisciplinary approach that integrates biological, psychological, and lifestyle-based strategies [302]. Collaborative care teams—comprising psychiatrists, psychologists, nutritionists, pharmacists, and exercise specialists—are well positioned to deliver comprehensive, personalized treatment plans [303]. Phytochemical supplementation can be particularly valuable when embedded within this broader therapeutic framework [304]. Nutritionists can guide dietary modifications that reinforce the anti-inflammatory and antioxidant actions of phytocompounds, while exercise specialists can prescribe physical activity regimens that synergize with phytochemicals to enhance mitochondrial health and neuroplasticity [305]. Psychologists can provide cognitive–behavioral or mindfulness-based therapies that further modulate stress-related pathways implicated in MDD [306]. The literature from integrated care models demonstrates that combining pharmacological and non-pharmacological interventions—such as in collaborative care or lifestyle psychiatry frameworks—improves clinical outcomes, enhances patient satisfaction, and reduces relapse rates [307]. For example, interventions that pair dietary polyphenols with structured aerobic exercise and therapy have shown additive effects on depressive symptom reduction and cognitive function [308]. This integrated model not only reflects the multifactorial nature of depression but also offers a pragmatic pathway to improve treatment adherence, reduce polypharmacy, and promote long-term mental wellness [309]. Phytochemicals, when coordinated with other interventions, may serve as powerful components of such a multidimensional care strategy.

This review illuminates the multifaceted nature of MDD and the potential utility of phytocompounds in its management. Recent evidence reveals that MDD involves not only monoaminergic dysregulation but also neuroinflammatory processes, oxidative stress, and mitochondrial dysfunction, implicating diverse pathophysiological pathways [223]. By evaluating interventional studies centered on phytochemicals—including polyphenols, flavonoids, and alkaloids—this review highlights their potential neuroprotective, anti-inflammatory, and antioxidant properties [22]. These effects align with emerging translational research suggesting that multi-targeted strategies may offer enhanced therapeutic outcomes in complex mood disorders [79,310]. Nevertheless, the heterogeneity of study designs, phytocompound formulations, and patient populations underscores the need for more robust trials with standardized protocols [311,312,313]. This discussion aims to synthesize the mechanistic underpinnings and clinical implications of phytocompound use in MDD, situating these findings within broader neuropsychiatric research and outlining key directions for future investigation. Taken together, these insights reaffirm the value of phytochemicals as complementary or alternative therapies in MDD, particularly given their favorable safety profiles, multimodal mechanisms of action, and promise for treatment-resistant populations. Continued clinical exploration of these natural interventions may significantly enhance holistic, personalized approaches to depression care and expand the future of integrative psychiatric treatment.

## 10. Conclusions

Emerging data suggest that specific plant-derived compounds can influence the neuro–immune interface and mitochondrial health in major depressive disorder, yet these signals remain preliminary. Wide variation in extract standardization, dosing protocols, study designs, and participant profiles hampers direct comparison, and most trials enroll modest, geographically limited samples. To translate early promise into clinical practice, large phase-III studies with rigorous pharmacokinetic assessment, active comparators, and extended safety follow-up are essential. Nonetheless, the literature reviewed here highlights the distinctive neuroprotective, anti-inflammatory, and antioxidant capacities of phytochemicals—attributes that align well with the complex biology of depression, extending therapeutic thinking beyond monoamine modulation alone. Although several investigations report meaningful symptom relief with minimal adverse effects, methodological shortcomings—small cohorts, heterogeneous interventions, and inconsistent endpoints—preclude definitive guidance. Future research must emphasize harmonized formulations, adequately powered populations, and mechanistic biomarkers to clarify durability, optimal dosing, and synergy with standard antidepressants. Collectively, the current evidence positions phytocompounds as promising adjuncts, warranting continued, methodologically robust exploration to fully harness their translational potential.

## Figures and Tables

**Table 1:** Summary of Interventional Studies Evaluating the Therapeutic Effects of Phytocompounds in Major Depressive Disorder (MDD). This table presents a structured synthesis of the 19 clinical trials included in the systematic review, selected according to PRISMA guidelines. Each study is characterized by its design, geographic location, sample demographics, intervention type, control conditions, outcome measures, and reported adverse events. The interventions include a range of phytocompounds—such as curcumin, flavonoids, anthocyanins, and isoflavones—administered in monotherapy or adjunctive formats. Outcomes were assessed via validated psychometric scales and, where applicable, supported by biochemical and neurotrophic markers. This compilation provides a comparative overview of study quality, efficacy, and safety in the clinical application of phytochemicals for MDD.

Study [Ref.]	Country	Population	Intervention	Outcomes	Side Effects
Soltani et al. [257]	Iran	42 patients (CSFP) aged 35–70	Nano-curcumin 80 mg/day, 12 weeks	Improved depression and quality of life	Nausea, headache, and diarrhea
Dehghani et al. [258]	Iran	76 individuals aged 35–65	Quercetin 500 mg/day, 8 weeks	Marginal depression improvement	Headache, joint pain, and abdominal discomfort
Hajiluian et al. [259]	Iran	50 MS patients with depression, aged 18–55	Ellagic acid 180 mg/day, 12 weeks	Reduced inflammation and depression	None significant
Choi et al. [263]	Republic of Korea	40 young adults aged 18–29 with MDD	Flavonoid-rich orange juice, 8 weeks	Significant improvement in depressive scores	None significant
Maeda-Yamamoto et al. [264]	Japan	15 older adults aged 50–70	Anthocyanin-rich potatoes, 8 weeks	Improved psychological stress response	None significant
Parilli-Moser et al. [265]	U.K.	38 new mothers	High-flavonoid diet, 2 weeks	Reduced anxiety and improved social relationships	None significant
Barfoot et al. [266]	Spain	63 young overweight adults	Roasted peanuts and peanut butter, 6–7 months	Significant depression reduction	Digestive symptoms and softer stools
Bourdel-Marchasson et al. [267]	Europe	125 elderly adults aged ~70	Antioxidant-rich diet, 2 months	Reduced depressive symptoms	None serious
Kontogianni et al. [260]	U.K.	99 mildly hypertensive adults aged 40–65	High vs. low polyphenol diet, 8 weeks	Improved depressive symptoms	Not reported
Park et al. [268]	Republic of Korea	40 healthy adults (20–30 years old)	Flavonoid-rich (FR) or flavonoid-low (FL) drinks, 190 mL twice daily for 8 week	↓ Depressive symptoms (CES-D < 20)	None significant
Smetanka et al. [269]	Slovakia	67 adults with MDD, aged 18–65	Pycnogenol with escitalopram, 12 weeks	No additional benefit compared to escitalopram alone	None significant
Kanchanatawan et al. [270]	Multinational	61 adults aged 18–63 with MDD history	Curcumin 500–1500 mg/day, 12–16 weeks	Improved depression scores (MADRS)	Dizziness, nausea, insomnia, and diarrhea
Terauchi et al. [261]	Italy	60 menopausal women aged 50–55	Equol and resveratrol, 12 weeks	Reduced depressive symptoms (HAM-D)	Mild diarrhea
Khalid et al. [271]	U.K.	Young adults and children	Wild blueberry drink (flavonoids), acute administration	Improved positive affect shortly after consumption	None significant
Hirose et al. [262]	Japan	87 menopausal women aged 40–60	Isoflavone aglycones, 8 weeks	Improved anxiety and modest depression changes	None significant
Sathyapalan et al. [272]	U.K., Iran	30 obese adults	Curcumin 1 g/day, 30 days	Reduced anxiety and no significant depression improvement	None significant
Terauch et al. [273]	Japan	91 menopausal women (40–60 years old)	GSPE (100 or 200 mg/day) vs. placebo, 8 weeks	↓ Anxiety (dose-dependent); no effect on depression	None significant
Pase et al. [11]	Australia	Adults aged 40–65	Cocoa polyphenols 250–500 mg/day, 30 days	Improved mood and calmness	None
Clinical pilot [274]	U.K.	10 adults	High-polyphenol chocolate, crossover	Improved anxiety and depression scores	None

AE: adverse events; AIS: Athens Insomnia Scale; BAI: Beck Anxiety Inventory; BDI: Beck Depression Inventory; BDI-II: Beck Depression Inventory-II; BDNF: brain-derived neurotrophic factor; CB: control butter; CES-D: Center for Epidemiological Studies Depression Scale; EDSS: expanded disability status scales; ESC: escitalopram; FL: low-flavonoid orange cordial group; FR: group of orange juice rich in flavonoids; GSPE: grape seed proanthocyanidin extract; HADS: Hospital Anxiety and Depression Scale; HAMD/HAMD -17: Hamilton Depression Rating Scale; IFN-γ: gamma interferon; IQR: range between quartiles; MADRS: Montgomery–Asberg Depression Rating Scale; MDD: major depressive disorder; MSC: mesenchymal stem cells; MSS: Menopause Symptom Scale; MS: multiple sclerosis; NO: nitric oxide; PA: positive affect; PB: peanut butter; PR: time interval between the beginning of atrial depolarization and the beginning of ventricular depolarization; PYC: pycnogenol; QRS: depolarization of the ventricles, consisting of Q, R, and S waves; SQ: Shadow Queen; SRP: roasted peanuts in shell; WBB: wild blueberry drink.

## Abbreviations

The following abbreviations are used in this manuscript:

BDNF	Brain-derived neurotrophic factor
CNS	Central nervous system
DNA	Deoxyribonucleic acid
HAM-D	Hamilton depression rating scale
IL	Interleukin
MMA	Methylmalonic acid
MDD	Major depressive disorder
mtDNA	Mitochondrial deoxyribonucleic acid
NF-κB	Nuclear factor kappa B
ROS	Reactive oxygen species
SSRI	Selective serotonin reuptake inhibitor
TCA	Tricyclic antidepressant
